# Biocatalytic Synthesis of Chiral Alcohols and Amino Acids for Development of Pharmaceuticals

**DOI:** 10.3390/biom3040741

**Published:** 2013-10-02

**Authors:** Ramesh N. Patel

**Affiliations:** SLRP Associates Consultation in Biotechnology, 572 Cabot Hill Road, Bridgewater, NJ 08807, USA; E-Mail: rameshpatelphd@yahoo.com; Tel.: +1-908-725-5738

**Keywords:** biocatalysis, enzymatic processes, chiral intermediates, drugs development

## Abstract

Chirality is a key factor in the safety and efficacy of many drug products and thus the production of single enantiomers of drug intermediates and drugs has become increasingly important in the pharmaceutical industry. There has been an increasing awareness of the enormous potential of microorganisms and enzymes derived there from for the transformation of synthetic chemicals with high chemo-, regio- and enatioselectivities. In this article, biocatalytic processes are described for the synthesis of chiral alcohols and unntural aminoacids for pharmaceuticals.

## 1. Introduction

For preparation of drugs and their intermediates, the synthesis of single enantiomers has become increasingly important in the pharmaceutical industry [1]. Single enantiomers can be produced by either by chemical or biocatalytic routes. The advantages of biocatalysis over chemical synthesis are that enzyme-catalyzed reactions are often highly enantioselective and regioselective. They can be carried out under mild conditions at ambient temperature and atmospheric pressure, thus avoiding the use of more extreme reaction conditions which could cause problems with isomerization, racemization, epimerization, and rearrangement of compound. Microbial cells and wide variety and class of enzymes derived there from can be used for chiral synthesis. Enzymes can be immobilized and reused for many cycles. In addition, enzymes can be over expressed to make biocatalytic processes economically efficient, and enzymes with modified activity can be tailor-made. Directed evolution of biocatalysts can lead to increased enzyme activity, selectivity and stability [2,3,4,5,6,7,8,9,10,11,12,13,14,15]. A number of review articles [16,17,18,19,20,21,22,23,24,25,26,27,28,29,30,31] have been published on the use of enzymes in organic synthesis. This chapter provides some examples of the use of enzymes for the synthesis chiral alcohols, unnatural amino acids, and amines for synthesis of phamaceuticals.

## 2. Enzymatic Preparation of Chiral Alcohols

### 2.1. Hydroxy Buspirone (Antianxiety Drug): Enzymatic Preparation of 6-Hydroxybuspirone

**Figure 1:**
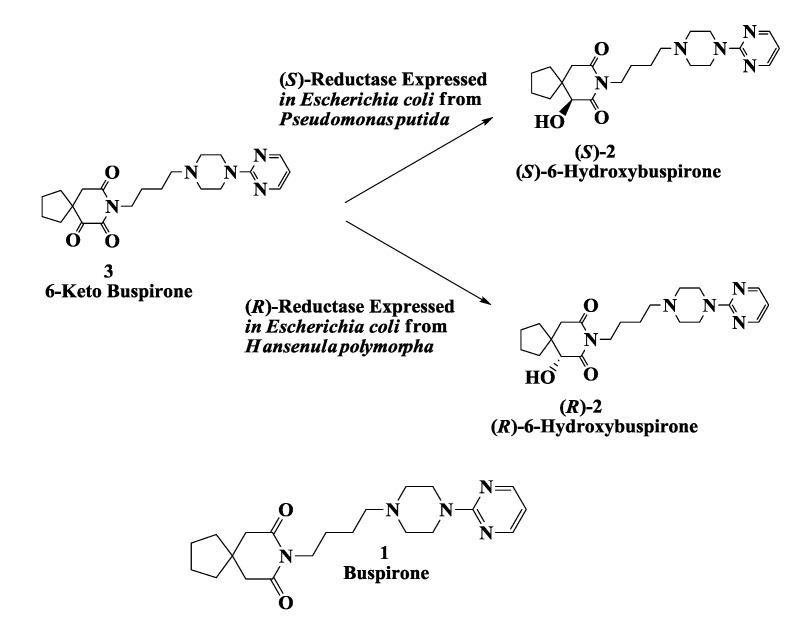
Hydroxy buspirone (antianxiety drug): Enzymatic preparation of 6-hydroxybuspirone.

Buspirone (Buspar^®^, **1**, Figure 1) is a drug used for treatment of anxiety and depression that is thought to produce its effects by binding to the serotonin 5HT1A receptor [32,33,34]. Mainly as a result of hydroxylation reactions, it is extensively converted to various metabolites and blood concentrations return to low levels a few hours after dosing [35]. A major metabolite, 6-hydroxybuspirone **2**, produced by the action of liver cytochrome P450 CYP3A4, is present at much higher concentrations in human blood than buspirone itself. For development of 6-hydroxybuspirone as a potential antianxiety drug, preparation and testing of the two enantiomers as well as the racemate was of interest. An enantioselective microbial reduction process was developed for reduction of 6-oxobuspirone **3**, to either (*R*)- and (*S*)-6-hydroxybuspirone **2**. About 150 microbial cultures were screened for the enantioselective reduction of **3**. *Rhizopus stolonifer* SC 13898, *Neurospora crassa* SC 13816, *Mucor racemosus* SC 16198, and *Pseudomonas putida* SC 13817 gave >50% reaction yields and >95% e.e.s of (*S*)-6-hydroxybuspirone. The yeast strains *Hansenula polymorpha* SC 13845 and *Candida maltosa* SC 16112 gave (*R*)-6-hydroxybuspirone **2** in >60% reaction yield and >97% e.e. [36]. The NADP-dependent (*R*)-reductase (RHBR) from *Hansenula polymorpha* SC 13845 was purified to homogeneity, its N-terminal and internal sequences were determined and cloned and expressed in *Escherichia coli*. To regenerate the cofactor NADPH required for reduction we have also cloned and expressed the glucose-6-phosphate dehydrogenase gene from *Saccharomyces cerevisiae* in *Escherichia coli*. Recombinant cultures expressing (*R*)-reductase (RHBR) catalyzed the reduction of 6-ketobuspirone to (*R*)-6-hydroxybuspirone in 99% yield and 99.9% e.e. at 50 g/L substrate input [37].

The NAD-dependent (*S*)-reductase (SHBR) from *Pseudomonas putida* SC 16269 was also purified to homogeneity, its N-terminal and internal sequences were determined and cloned and expressed in *Escherichia coli*. To regenerate the cofactor NADH required for reduction we have also cloned and expressed the NAD^+^ dependent formate dehydrogenase gene from *Pichia pastoris* in *Escherichia coli*. Recombinant *Escherichia coli* expressing (*S*)-reductase was used to catalyze the reduction of 6-ketobuspirone to (*S*)-6-hydroxybuspirone, in >98% yield and >99.8% e.e. at 50 g/L substrate input [37].

### 2.2. Cholesterol Lowering Agents: Enzymatic Preparation of (3S,5R)-Dihydroxy-6-(Benzyloxy) Hexanoic Acid, Ethyl Ester 4

**Figure 2:**
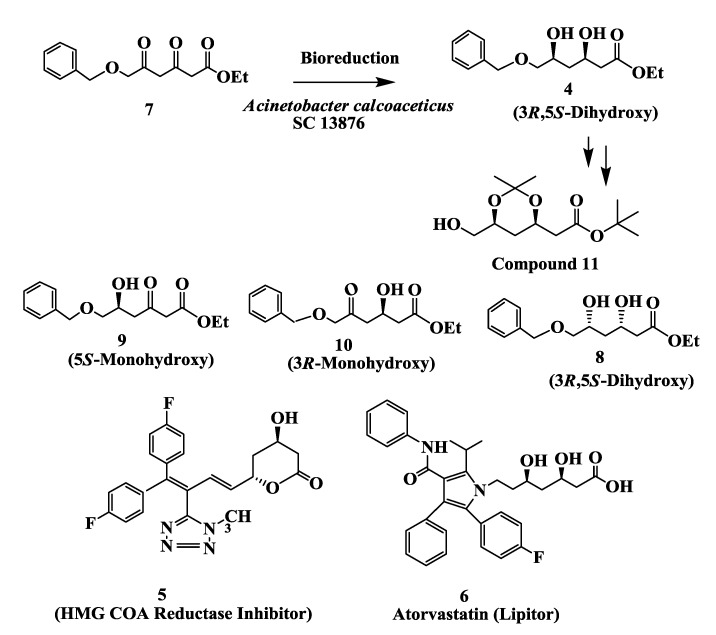
Cholesterol lowering agents: Enzymatic preparation of (3*S*,5*R*)-dihydroxy-6- (benzyloxy) hexanoic acid, ethyl ester.

Compound **4** (Figure 2) is a key chiral intermediate required for the chemical synthesis of compound **5**, Arotvastatin **6**, and Rosuvastatin all are anticholesterol drugs which acts by inhibition of HMG CoA reductase [38,39,40,41,42]. 

The enantioselective reduction of a diketone 3,5-dioxo-6-(benzyloxy) hexanoic acid, ethyl ester **7** to (3*R*,5*S*)-dihydroxy-6-(benzyloxy) hexanoic acid, ethyl ester **4** (Figure 2) was demonstrated by cell suspensions of *Acinetobacter calcoaceticus* SC 13876 [39,43]. On reduction of **7** by cell suspensions, the *syn*-**4** and *anti*-**8** dihydroxy esters were formed in the ratio of about 87:13, 83:17, 76:24 after 24 h at 2, 5 and 10 g/L of substrate input, respectively. There was no significant peak due to a monohydroxy ester. Chiral HPLC determined that the desired (3*R*,5*S*)-**4** was the major product with 99.4% e.e. Almost complete (>95%) conversion of the ethyl diketoester **7** to dihydroxy ester **4** in 24 h was seen up to a substrate concentration of 10 g/L and cell concentration of 200 g/L [39,43].

A mixture of ethyl 3-keto-5-hydroxy **9** (major) and 5-keto-3-hydroxy **10** (minor) was obtained from partial microbial reduction of ketoester **7**. These two mixtures were subjected to microbial reduction by *Acinetobacter sp* SC13874 cells for 6 h (incomplete reduction). The reduction provided the dihydroxy esters with the isomeric composition. The results indicated that the second reduction of the monohydroxy compound by SC13874 cells was quite enantiospecific. Reduction of the 3-keto-5-hydroxy **9** provided predominantly the (3*R)-*hydroxy, while reduction of the 3-hydroxy-5-keto ester **10** provided predominantly the (5*S)*-hydroxy compound [43].

Cell extracts of A. *calcoaceticus* SC 13876 in the presence of NAD^+^, glucose, and glucose dehydrogenase reduced **7** to the corresponding monohydroxy compounds [3-hydroxy-5-oxo-6-(benzyloxy) hexanoic acid ethyl ester 9 and 5-hydroxy-3-oxo-6-(benzyloxy) hexanoic acid ethyl ester 10]. Both **9** and **10** were further reduced to the (3*R*,5*S*)-dihydroxy compound **4** in 92% yield and 99% e.e. by cell extracts. (3*R*,5*S*)-**4** was converted to **11**, a key chiral intermediate for the synthesis of compound **5** and Atorvastatin **6**. Three different ketoreductases were purified to homogeneity from cell extracts of *A. calcoaceticus* SC 13876 and their biochemical properties were compared. Reductase I only catalyzes the reduction of ethyl diketoester **7** to its monohydroxy products whereas reductase II catalyzes the formation of dihydroxy products from monohydroxy substrates. A third reductase (III) was identified which catalyzes the reduction of diketoester **7** to *syn*-(3*R*,5*S*)-dihydroxy ester **4** [44], which now has been cloned and expressed in *E. coli* [44] and the reduction of diketoester 7 to *syn*-(3*R*,5*S*)-dihydroxy ester **4** was demonstrated by recombinant enzyme at 50 g/L substrate input with 10 g/L cell suspensions.

### 2.3. Atorvastatin: Enzymatic Preparation of (R)-4-Cyano-3-Hydroxybutyrate

**Figure 3:**
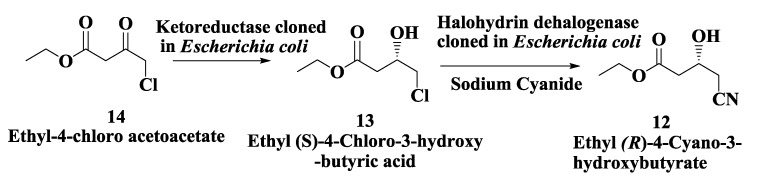
Atorvastatin: Enzymatic preparation of (*R*)-4-cyano-3-hydroxybutyrate.

An enzymatic process for the preparation of ethyl (*R*)-4-cyano-3-hydroxybutyric acid **12** (Figure 3), a key intermediate for the synthesis of Atorvastatin **6** was developed by Codexis [45]. In this process, first the enzymatic synthesis of ethyl (*S*)-4-chloro-3-hydroxybutyric acid derivatives **13** was carried out by ketoreductase-catalyzed conversion of 4-chloro-3-ketobutyric acid derivatives **14** [46]. The genes encoding halohydrin dehydrogenase from *Agrobacterium tumefaciens*, ketoreductase from *Candida magnoliae*, glucose dehydrogenase from *Bacillus subtilis* and formate dehydrogenase from *Candida boidinii* were separately cloned into *Escherichia coli* BL21. Each enzyme was then produced by fermentation, isolated and characterized. Then ethyl (*R*)-4-cyano-3-hydroxybutyrate **12** (Figure 3) was prepared from ethyl 4-chloroacetoacetate **14** by the following procedure: Ethyl 4-chloroacetoacetate 14 was incubated at pH 7.0 with ketoreductase, glucose dehydrogenase, and NADP^+^ for 40 h to produce ethyl (*S*)-4-chloro-3-hydroxybutyrate **13** which was extracted with ethyl acetate, dried, filtered and concentrated to yield ~97% pure ester. The dried ethyl (*S*)-4-chloro-3-hydroxybutyrate **13** was dissolved in phosphate buffer and mixed with halohydrin dehalogenase and sodium cyanide at pH 8.0. After 57 h, essentially pure ethyl (*R*)-4-cyano-3-hydroxybutyrate **12**, an intermediate used in HMG-CoA reductase inhibitors syntheses, was recovered [45].

### 2.4. Preparation of (S)-4-Chloro-3-Hydroxybutanoic Acid Methyl Ester

**Figure 4:**
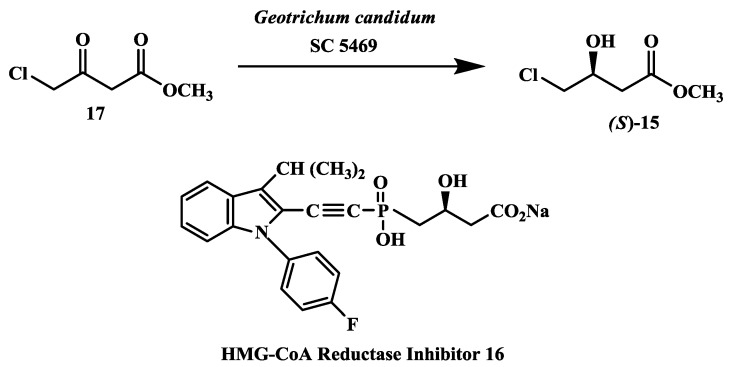
Chloesterol lowering agents: Preparation of (*S*)-4-chloro-3-hydroxybutanoic acid methyl ester.

**(***S*)-4-chloro-3-hydroxybutanoic acid methyl ester **15** (Figure 4) is a key chiral intermediate in the total chemical synthesis of **16**, an inhibitor of HMG CoA reductase [46,47]. The reduction of 4-chloro-3-oxobutanoic acid methyl ester **17** to (*S*)-4-chloro-3-hydroxybutanoic acid methyl ester **15** (Figure 4) by cell suspensions of *Geotrichum candidum* SC 5469. In the biotransformation process, a reaction yield of 95% and e.e. of 96% were obtained for (*S*)-**15** by glucose-, acetate- or glycerol-grown cells (10% w/v) of *G. candidum* SC 5469 at 10 g/L substrate input. The e.e. of (*S*)-**15** was increased to 98% by heat-treatment of cell-suspensions (55 °C for 30 min) prior to conducting the bioreduction of **17** [48].

In an alternate approach, the asymmetric reduction of ethyl 4-chloroacetoacetate to (*S*)-4-chloro-3-hydroxybutonoate was demonstrated by a secondary alcohol dehydrogenase (PfODH) from *Pichia finlandica*. The gene encoding PfODH was cloned from *P. finlandica* and over expressed in *Escherichia coli*. Formate dehydrogenase was used to regenerate the cofactor NADH required for this reaction. Using recombinant *E. coli* coexpressing both PfODH and formate dehydrogenase from *Mycobacetrium* sp. produced to (*S*)-4-chloro-3-hydroxybutonoate in 98.5% yield and 99% e.e. at 32 g/L substrate input [49].

### 2.5. Rhinovirus Protease Inhibitor: Enzymatic Process for the Preparation of (R)-3-(4-Fluorophenyl)-2-Hydroxy Propionic Acid

**Figure 5:**
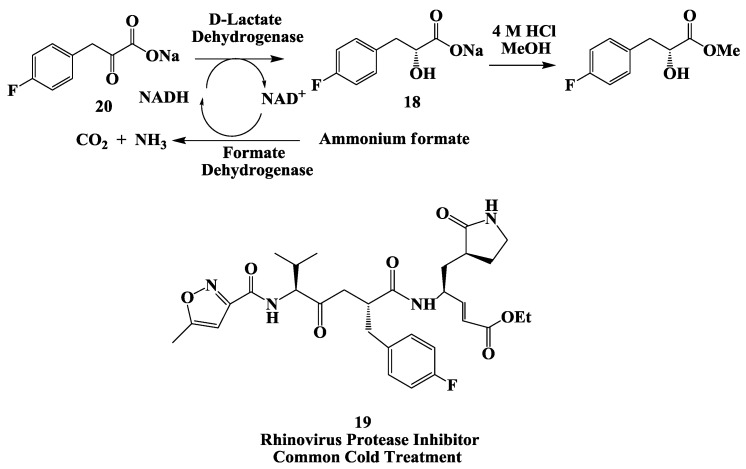
Rhinovirus protease inhibitor: Enzymatic process for the preparation of (*R*)-3-(4-fluorophenyl)-2-hydroxy propionic acid.

(*R*)-3-(4-fluorophenyl)-2-hydroxy propionic acid **18** (Figure 5) is a building block for the synthesis of AG7088, a rhinovirus protease inhibitor **19** [50,51]. The preparation of **18** using a biocatalytic reduction of **20** in a membrane reactor [52]. A continuous enzymatic process for an efficient synthesis of (*R*)-3-(4-fluorophenyl)-2-hydroxy propionic acid at multikilogram scale with a high space-time yield (560 g/L/day) using a membrane reactor. The product was generated in excellent enantiomeric excess (e.e. > 99.9%) and good overall yield (68%–72%).

Using this method, an overall quantity of 23 kg of **18** was prepared. The key step was an aqueous enzymatic reduction using D-lactate dehydrogenase (D-LDH) and formate dehydrogenase (FDH). Mechanistically, the keto acid salt **20** is stereoselectively reduced to the corresponding *R*-hydroxy acid **18** in the presence of D-lactate dehydrogenase by NADH. The cofactor itself is oxidized to NAD^+^ in the process. Subsequently, in the presence of formate dehydrogenase, NAD^+^ is reduced back to NADH by ammonium formate, which was oxidized to CO_2_ and NH_3_. In this fashion the expensive cofactor NAD^+^ is regenerated by FDH, and only a catalytic amount of NAD^+^ was required [52].

### 2.6. Enzymatic Preparation of Chiral Intermediates for Atazanavir

**Figure 6:**
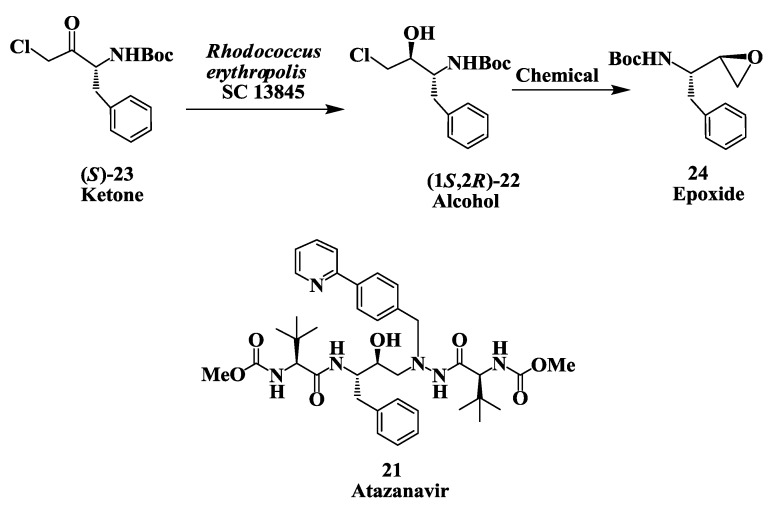
Atazanavir (antiviral agent): Enzymatic reparation of (1*S*,2*R*)-[3-chloro-2-hydroxy-1-(phenylmethyl) propyl]-carbamic acid,1,1-dimethyl-ethyl ester.

Atazanavir **21** (Figure 6) is an acyclic aza-peptidomimetic, a potent HIV protease inhibitor [53,54] approved by the Food and Drug Administration for treatment of Auto Immune Diseases (AIDS). An enzymatic process was developed for the preparation of (1*S*,2*R*)-[3-chloro-2-hydroxy-1-(phenylmethyl) propyl]carbamic acid, 1,1-dimethylethyl ester **22** , a key chiral intermediate required for the total synthesis of the HIV protease inhibitor atazanavir. The diastereoselective reduction of (1*S*)-[3-chloro-2-oxo-1-(phenylmethyl)propyl] carbamic acid, 1,1-dimethylethyl ester **23** was carried out using *Rhodococcus*, *Brevibacterium*, and *Hansenula* strains to provide **22**. Three strains of *Rhodococcus* gave >90% yield with a diastereomeric purity of >98% and an e.e. of 99.4% [55]. An efficient single-stage fermentation-biotransformation process was developed for the reduction of ketone **23** with cells of *Rhodococcus erythropolis* SC 13845 to yield **22** in 95% with a diasteromeric purity of 98.2% and an e.e. of 99.4% at substrate input of 10 g/L. The reduction process was further improved by generating mutants and selection of desired mutant for conversion of **23** to (1*S*,2*R*)-**22** at substrate input of 60 g/L [56]. (1*S*,2*R*)-22 was converted to epoxide **24** and used in the synthesis of atazanavir. Chemical reduction of chloroketone **23** using NaBH_4_ produces the undesired chlorohydrin diastereomer [57]. 

### 2.7. Enzymatic Reduction Process for Synthesis of Montelukast Intermediate

**Figure 7:**
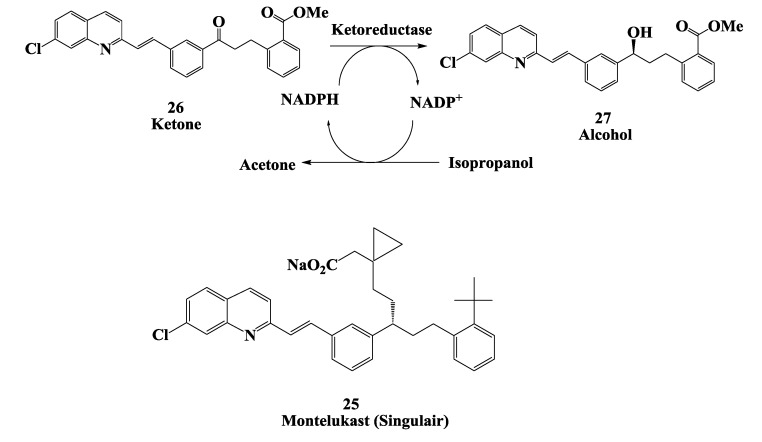
Enzymatic reduction process for synthesis (*S*)-alcohol **27** for Montelukast intermediate.

The discovery of the biological activity of the slow reacting substance of anaphylaxis (SRS-A) and its relation to the leukotrienes (LTC4, LTD4, and LTE4) and asthma, the search for leukotriene antagonists has been intensive. As part of an ongoing program for the development of specific LTD4 antagonists for the treatment of asthma and other associated diseases at Merck have identified Montelukast **25** (Figure 7) as LTD4 antagonist [58,59,60].

Merck has described the synthetic route for the production of montelukast, using a stereoselective reduction of a ketone **26** to the (*S*)-alcohol **27** as the key step. The alcohol subsequently undergoes a Sn2 displacement with a thiol to give the *R*-configured final product [59,60]. The reduction of the ketone **26** to produce the chiral alcohol **27** requires stoichiometric amounts of the chiral reducing agent (−)-flchlorodiisopino campheylborane [(−)-DIP-chloride]. (−)-DIP-chloride is selective and avoids the side reactions but it is corrosive and moisture-sensitive, causing burns if it is allowed to contact the skin. The reaction must be carried out at −20 to −25 °C to achieve the best stereoselectivity. The quench and extractive work-up generate large volumes of waste solvent, due to the product’s low solubility. The potential advantages of biocatalytic transformation of ketone to alcohol were recognized early on by researchers at Merck. However, only two microorganisms were identified as having activity on the bulky and hydrophobic substrate [61]. Due to several reasons, an enzyme-catalyzed process for reduction of the ketone **26** was developed by Codexis. A ketoreductase was developed by directed evolution by high throughput screens using a slurry of the ketone substrate and high isopropanol concentration. Beneficial mutations among the various improved mutants were recombined in each round, and new mutations were made guided by ProSAR. The productivity of the final enzyme was improved 2,000-fold and stability was also substantially increased [62].

The final process was carried out as a slurry-to-slurry reaction at 45 °C, with the sparingly soluble ketone **26** being converted to an almost equally insoluble alcohol **27** at a concentration of 100 g/L substrate in aqueous isopropanol and toluene. A reaction yield of 99.3% and enantiomeric excess of 99.9% was obtained for alcohol 27 [62].

### 2.8. Anticancer Drug: Enzymatic Preparation of C-13 Paclitaxel Side-Chain Synthon

**Figure 8:**
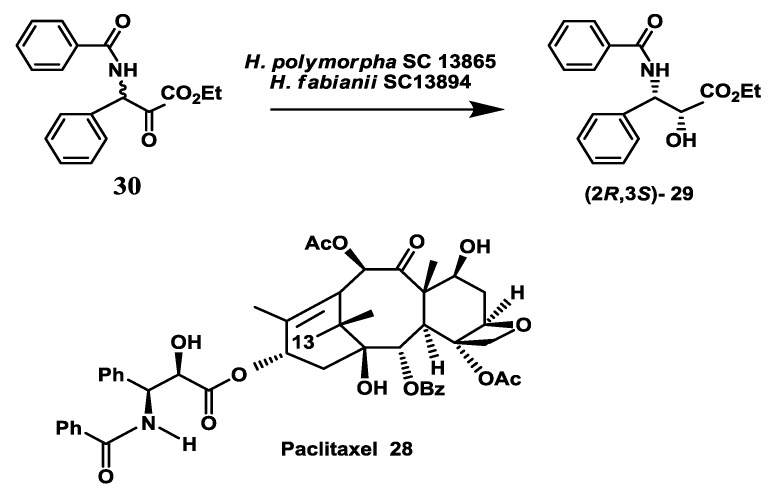
Anticancer drug: Enzymatic preparation of C-13 paclitaxel side-chain synthon.

Among the antimitotic agents, paclitaxel (taxol®) **28** (Figure 8), a complex, polycyclic diterpene, exhibits a unique mode of action on microtubule proteins responsible for the formation of the spindle during cell division. Various types of cancers have been treated with paclitaxel and it was approved for use by the FDA for treatment of ovarian cancer and metastatic breast cancer [63,64,65]. A key precursor for the paclitaxel semi-synthetic process is the chiral C-13 paclitaxel side-chain **29**. An enzymatic enantioselective microbial reduction of 2-keto-3-(N-benzoylamino)-3-phenyl propionic acid ethyl ester **30** to yield (2*R*,3*S*)-*N*-benzoyl-3-phenyl isoserine ethyl ester **29** was demonstrated using two strains of *Hansenula* [66]. Preparative-scale bioreduction of ketone **30** was demonstrated using cell suspensions of *Hansenula polymorpha* SC 13865 and *Hansenula fabianii* SC 13894 in independent experiments. In both batches, a reaction yield of >80% and e.e.s of >94% were obtained for (2*R*,3*S*)-**29**. In a single-stage process, cells of *H. fabianii* were grown in a 15-L fermentor for 48 h, then the bioreduction process was initiated by addition of 30 g of substrate and 250 g of glucose and continued for 72 h. A reaction yield of 88% with an e.e. of 95% was obtained for (2*R*,3*S*)-**29**.

### 2.9. Antipsychotic Drug: Enzymatic Reduction of 1-(4-Fluorophenyl)4-[4-(5-Fluoro-2-Pyrimidinyl)1-Piperazinyl]-1-Butanone

**Figure 9:**
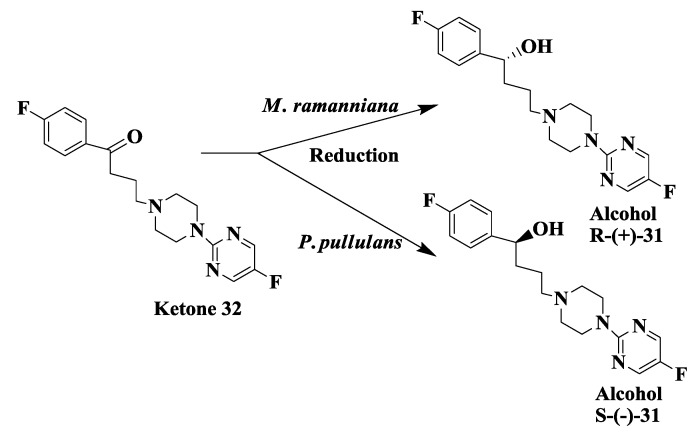
Antipsychotic drug: Enzymatic reduction of 1-(4-fluorophenyl)4-[4-(5-fluoro-2-pyrimidinyl)1-piperazinyl]-1-butanone.

The sigma receptor system in the brain and endocrine tissue has been target for development of new class of antipsychotic drugs [67,68]. Compound (*R*)-**31** (Figure 9) is a sigma ligand and has a high affinity for sigma binding site and antipsychotic efficacy. The enantioselective microbial reduction process was developed for the conversion of ketone **32** to both enantiomers of alcohol **31** [69]. Various microorganisms screened for the enatioselective reduction of 1-(4-fluorophenyl)4-[4-(5-fluoro-2-pyrimidinyl)1-piperazinyl]-1butanone **32**. From this screen, *Mortierella ramanniana* ATCC 38191 was identified to predominantly reduced compound **32** to (*R*)-**31**, while *Pullularia pullulans* ATCC 16623 was identified to predominantly reduced compound **32** to (*S*)-**31**. A single stage fermentation/biotransformation process was developed. Cells of *M.* *ramanniana* were grown in a 20-L fermentor and after 40 h growth period, the biotransformation process was initiated by addition of 40 g ketone **32** and 400 g glucose. The biotransformation process was completed in 24 h with a reaction yield of 100% and an e.e. of 98.9% for (*R*)-**31**. At the end of the biotransformation process, cells were removed by filtration and product was recovered from the filtrate in overall 80% yield [69].

### 2.10. Retinoic Acid Receptpor Agonist: Enzymatic Preparation of 2-(R)-Hydroxy-2-(1',2',3',4'-Tetrahydro-1',1',4',4'-Tetramethyl-6'-Naphthalenyl)Acetate

**Figure 10:**
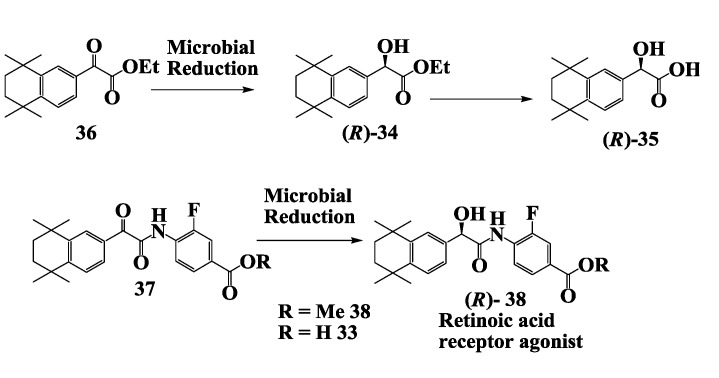
Retinoic acid receptor agonist: Enzymatic preparation of 2-(*R*)-hydroxy-2-(1',2',3',4'-tetrahydro-1',1',4',4'-tetramethyl-6'-naphthalenyl)acetate.

Retinoic acid and its natural and synthetic analogs (retinoids) exert a wide variety of biological effects by binding to or activating a specific receptor or sets of receptors [70]. They have been shown to effect cellular growth and differentiation and are promising drugs for the treatment of cancers [71]. A few retinoids are already in clinical use for the treatment of dermatological diseases such as acne and psoriasis. (*R*)-3-Fluoro-4-[[hydroxy-(5,6,7,8-tetrahydro-5,5,8,8-tetramethyl-2-naphthalenyl)-acetyl]amino]benzoic acid **33** (Figure 10) is a retinoic acid receptor gamma-specific agonist potentially useful as a dermatological and anticancer drug [72]. 

Ethyl 2-(*R*)-hydroxy-2-(1',2',3',4'-tetrahydro-1',1',4',4'-tetramethyl-6'-naphthalenyl)acetate **34** and the corresponding acid **35** were prepared as intermediates in the synthesis of the retinoic acid receptor gamma-specific agonist [73]. Enantioselective microbial reduction of ethyl 2-oxo-2-(1',2',3',4'-tetrahydro-1',1',4',4'-tetramethyl-6-naphthalenyl) acetate **36** to alcohol **34** was carried out using *Aureobasidium pullulans* SC 13849 in 98% yield and with an e.e. of 96%. At the end of the reaction, hydroxyester **34** was adsorbed onto XAD-16 resin and, after filtration, recovered in 94% yield from the resin with acetonitrile extraction. The recovered (*R*)-hydroxyester **34** was treated with Chirazyme L-2 or pig liver esterase to convert it to the corresponding (*R*)-hydroxyacid **35** in quantitative yield. The enantioselective microbial reduction of ketoamide **37** to the corresponding (*R*)-hydroxyamide **38** by *A. pullulans* SC 13849 has also been demonstrated [73]. 

### 2.11. Anti-Alzheimer’s Drugs: Enzymatic Reduction of 5-Oxohexanoate and 5-Oxohexanenitrile

**Figure 11:**
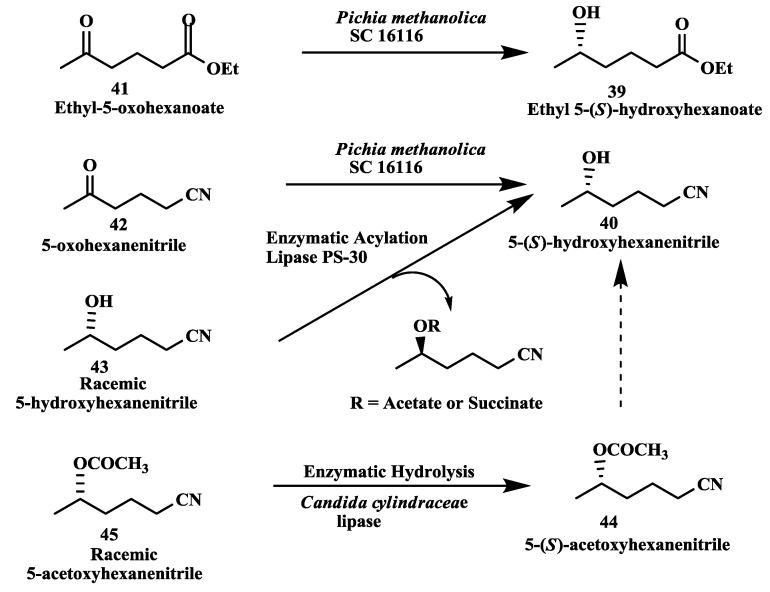
Anti-Alzheimer’s drugs: Enzymatic reduction of 5-oxohexanoate and 5-oxohexanenitrile.

Ethyl-(*S*)-5-hydroxyhexanoate **39** and (*S*)-5-hydroxyhexanenitrile **40** (Figure 11) are key chiral intermediates in the synthesis of anti-Alzheimer’s drugs [74]. Both chiral compounds have been prepared by enantioselective reduction of ethyl-5-oxohexanoate **41** and 5-oxohexanenitrile 42 by *Pichia methanolica* SC 16116 [75]. Reaction yields of 80%–90% and >95% e.e.s were obtained for each compound. In an alternate approach, the enzymatic resolution of racemic 5-hydroxyhexane nitrile **43** by enzymatic succinylation was demonstrated using immobilized lipase PS-30 to obtain (*S*)-5-hydroxyhexanenitrile **40** in 35% yield (maximum yield is 50%). (*S*)-5-Acetoxy-hexanenitrile **44** was prepared by enantioselective enzymatic hydrolysis of racemic 5-acetoxyhexanenitrile **45** by *Candida antarctica* lipase. A reaction yield of 42% and an e.e. of >99% were obtained [75].

### 2.12. Enantioselective Microbial Reduction of Substituted Acetophenone

**Figure 12:**
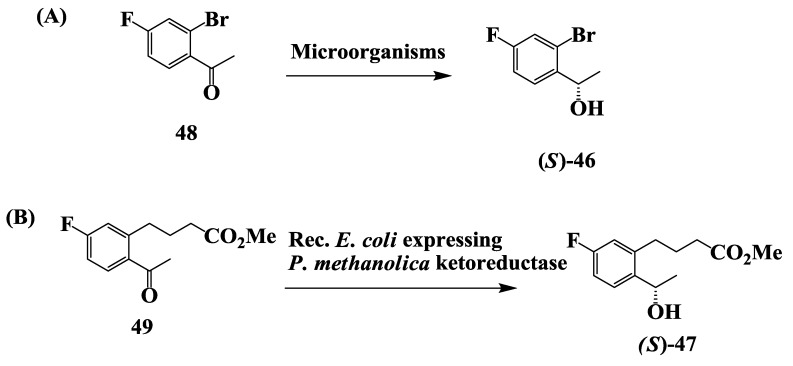
(**A**) Anti-Alzheimer’s drugs: Enantioselective microbial reduction of substituted acetophenone; (**B**) Enantioselective microbial reduction of methyl-4-(2'-acetyl-5'-fluorophenyl) butanoates.

The chiral intermediates (*S*)-1-(2'-bromo-4'-fluorophenyl)ethanol 46 and (*S*)-methyl 4-(2'-acetyl-5'-fluorophenyl)-butanol **47** are potential intermediates for the synthesis of several potential anti-Alzheimer’s drugs [76]. The chiral intermediate (*S*)-1-(2'-bromo-4'-fluoro phenyl)ethanol **46** (Figure 12A) was prepared by the enantioselective microbial reduction of 2-bromo-4-fluoro acetophenone **48** [77]. Organisms from genus *Candida*, *Hansenula*, *Pichia*, *Rhodotorula*, *Saccharomyces*, *Sphingomonas* and Baker’s yeast reduced **48** to **46** in >90% yield and 99% enantiomeric excess (e.e.). 

In an alternate approach, the enantioselective microbial reduction of methyl-4-(2'-acetyl-5'-fluorophenyl) butanoates **49** (Figure 12B) was demonstrated using strains of *Candida* and *Pichia*. Reaction yields of 40%–53% and e.e.s of 90%–99% were obtained for the corresponding (*S*)-hydroxy esters **47**. The reductase which catalyzed the enantioselective reduction of ketoesters was purified to homogeneity from cell extracts of *Pichia methanolica* SC 13825. It was cloned and expressed in *Escherichia coli* and recombinant cultures were used for the enantioselective reduction of the keto-methyl ester **49** to the corresponding (*S*)-hydroxy methyl ester **47**. On preparative scale, a reaction yield of 98% with an enantiomeric excess of 99% for **47** was obtained [77]. 

### 2.13. Anticancer Drug: Enzymatic Preparation of (S)-2-Chloro-1-(3-Chlorophenyl)Ethanol

**Figure 13:**
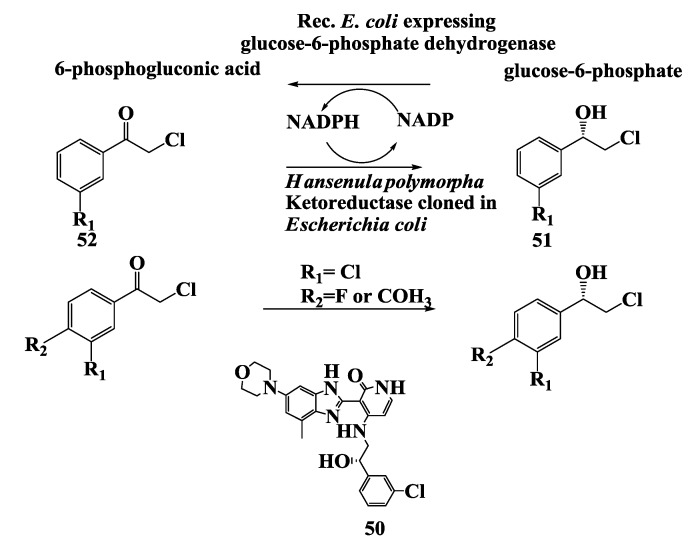
Anticancer drug: Enzymatic preparation of (*S*)-2-chloro-1-(3-chlorophenyl)ethanol.

The synthesis of the leading candidate compound **50** [78] in an anticancer program (IGF-1 receptor inhibitors) [79,80] required (*S)*-2-chloro-1-(3-chlorophenyl)ethanol **51** (Figure 13) as an intermediate. Other possible candidate compounds used are analogs of (*S*)-alcohol **51**. From microbial screen of the reduction of ketone **52** to (*S*)-alcohol **51**, two cultures namely *Hansenula polymorpha* SC13824 (73.8% enantiomeric excess) and *Rhodococcus globerulus* SC SC16305 (71.8% enantiomeric excess) were identified that had the highest enantioselectivity. A ketoreductase from *Hansenula polymorpha*, after purification to homogeneity, gave (*S*)-alcohol **51** with 100% ee [81]. The ketoreductase was cloned and expressed in *E. coli* together with a glucose-6-phosphate dehydrogenase from *Saccharomyces cerevisiae* to allow regeneration of the NADPH required for the reduction process. An extract of *E. coli* containing the two recombinant enzymes was used to reduce 2-chloro-1-(3-chloro-4fluorophenyl)ethanone **52**. Intact *E. coli* cells provided with glucose were used to prepare (*S)*-2-chloro-1-(3-chloro-4-fluorophenyl)ethanol **51** in 89% yield with 100% e.e. [81]. 

### 2.14. Thrombin Inhibitor: Enzymatic Preparation of (R)-2-Hydroxy-3,3-Dimethylbutanoic Acid

**Figure 14:**
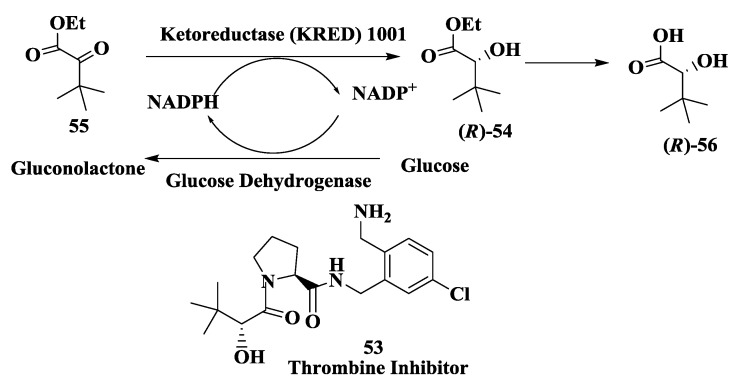
Thrombin inhibitor: Enzymatic preparation of (*R*)-2-Hydroxy-3,3-dimethylbutanoic acid.

Thrombin is a trypsin-like protease enzyme that plays a critical role in intrinsic and extrinsic blood coagulation. As a result of the enzymatic activation of numerous coagulation factors, thrombin is activated to cleave fibrinogen, producing fibrin, which is directly responsible for blood clotting. An imbalance between these factors and their endogenous activators and inhibitors can give rise to a number of disease states such as myocardial infarction, unstable angina, stroke, ischemia, restenosis following angioplasty, pulmonary embolism, deep vein thrombosis, and arterial thrombosis [82,83]. Consequently, the aggressive search for a potent, selective, and bioavailable thrombin inhibitor is widespread [84]. An intensive effort by Merck has led to the identification of thrombin inhibitor **53** [85]. The synthesis of **53** required a key chiral intermediate (*R*)-hydroxy ester **54**. An enzymatic process was developed for the asymmetric reduction of ketoester **55** to (*R*)-**54** using commercially available ketoreductase KRED1001 (Figure 14). The cofactor NADPH required for this reaction was regenerated using glucose dehydrogenase. The hydroxy ester (*R*)-**54** was isolated as an oil and then saponified to the corresponding enantiomerically pure hydroxy acid (*R*)-**56** without epimerization [86]. The enantiomerically pure (*R*)-**56** was obtained in 82% isolated yield (>99.5% e.e.).

### 2.15. Endothelin Receptor Antagonist: Enantioselective Microbial Reduction of Keto Ester and Chloroketone

**Figure 15:**
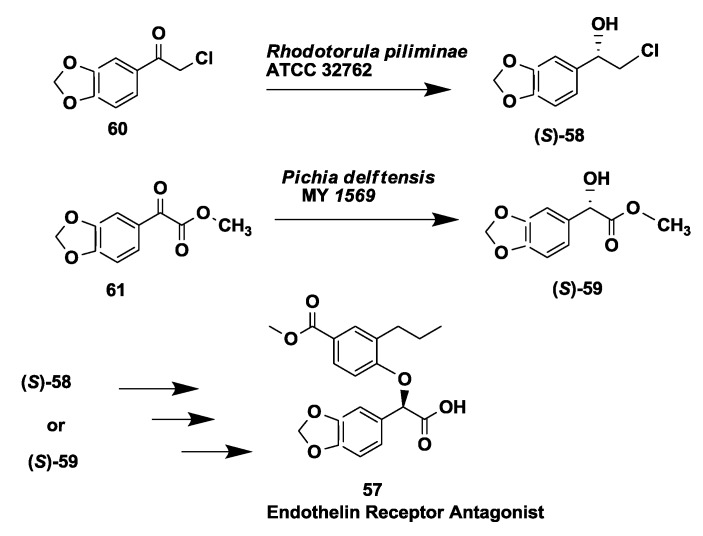
Endothelin receptor antagonist: Enantioselective microbial reduction of keto ester and chloroketone.

Endothelin is present in elevated levels in the blood of patients with hypertension, acute myocardial infarction and pulmonary hypertension. Two endothelin receptor sub-types have been identified which bind endothelin, thus causing vasoconstriction [87,88]. Endothelin receptor antagonists such as compound **57** (Figure 15) have potential therapeutic value. Synthesis of compound **57** required two key chiral intermediates (*S*)-alcohols **58** and **59**. Enantioselective microbial reduction of a ketoester **60** and a chlorinated ketone **61** to their corresponding (*S*)-alcohols **58** and **59** was demonstrated using *Pichia delftensis* MY 1569 and *Rhodotorula piliminae* ATCC 32762 to afford desired products in >98% e.e. and >99% e.e, respectively [89]. Reductions were scaled up to 23 L to produce the desired (*S*)-alcohols in 88% and 97% yields, respectively.

### 2.16. Calcium Channel Blocker: Preparation of [(3R-cis)-1,3,4,5-Tetrahydro-3-Hydroxy-4-(4-Methoxyphenyl)-6-(Trifluromethyl)-2H-1-Benzazepin-2-One]

**Figure 16:**
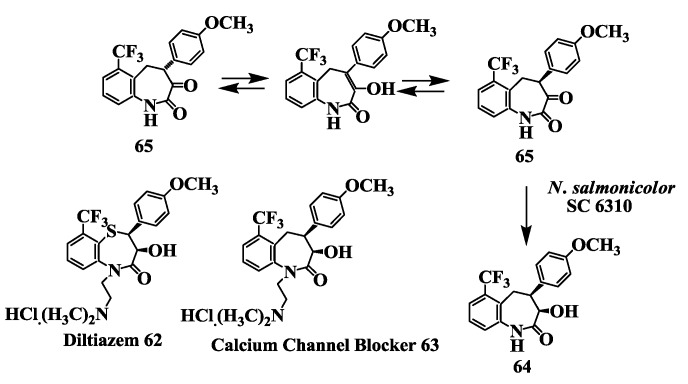
Calcium channel blocker: Preparation of [(3*R*-*cis*)-1,3,4,5-tetrahydro-3-hydroxy-4-(4-methoxyphenyl)-6-(trifluromethyl)-2*H*-1-benzazepin-2-one].

Diltiazem **62** (Figure 16) a benzothiazepinone calcium channel blocking agent that inhibits influx of extracellular calcium through L-type voltage-operated calcium channels, has been widely used clinically in the treatment of hypertension and angina [90]. Since diltiazem has a relatively short duration of action [91], an 8-chloro derivative recently has been introduced into the clinic as a more potent analogue [92]. Lack of extended duration of action and little information on structure-activity relationships in this class of compounds led Floyd *et al*. [93] to prepare isosteric 1-benzazepin-2-ones; this led to identification of (*cis*)-3-(acetoxy)-1-[2-(dimethylamino)ethyl]-1,3,4,5-tetrahydro-4-(4-methoxyphenyl)-6-trifluoromethyl)-2H-1-benzazepin-2-one **63** as a longer lasting and more potent antihypertensive agent. A key intermediate in the synthesis of this compound was (3*R*-*cis*)-1,3,4,5-tetrahydro-3-hydroxy-4-(4-methoxyphenyl)-6-(trifluromethyl)-2*H*-1-benzazepin-2-one **64**. An enantioselective process was developed for the reduction of 4,5-dihydro-4-(4-methoxyphenyl)-6-(trifluoromethyl)-1*H*-1-benzazepin-2,3-dione **65** to **64** using *Nocardia salmonicolor* SC 6310, in 96% reaction yield with 99.8% e.e. [94].

### 2.17. β3-Receptor Agonist: Reduction of 4-Benzyloxy-3-Methanesulfonylamino-2'-Bromo-Acetophenone

**Figure 17:**
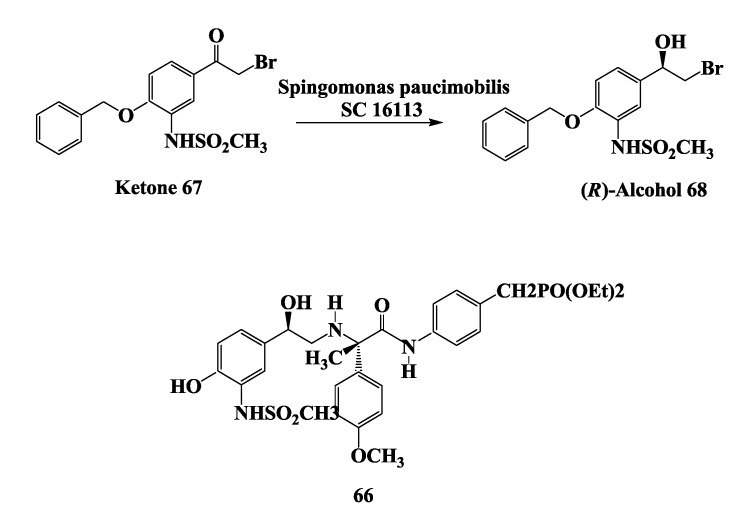
β3-Receptor agonist: Reduction of 4-benzyloxy-3-methanesulfonylamino-2-bromo-acetophenone.

β3-Adrenergic receptors are found on the cell surfaces of both white and brown adipocytes and are responsible for lipolysis, thermogenesis, and relaxation of intestinal smooth muscle [95]. Consequently, several research groups are engaged in developing selective β3 agonists for the treatment of gastrointestinal disorders, type II diabetes, and obesity [96,97]. Biocatalytic syntheses of chiral intermediates required for the total synthesis of β3 receptor agonists **66** (Figure 17) has been demonstrated [98]. 

The microbial reduction of 4-benzyloxy-3-methanesulfonylamino-2'-bromo-acetophenone **67** to the corresponding (*R*)-alcohol **68** has been demonstrated [98] using *Sphingomonas. paucimobilis* SC 16113. The growth of *S. paucimobilis* SC 16113 was carried out in a 750-L fermentor and harvested cells (60 kg) were used to conduct the biotransformation in 10-L and 200-L preparative batches using 20% (wt/vol, wet cells). In some batches, the fermentation broth was concentrated 3-fold by microfilteration and subsequently washed with buffer by diafilteration and used directly in the bioreduction process. In all the batches, reaction yields of >85% and e.e.s. of >98% were obtained. The isolation of alcohol **68** from the 200-L batch gave 320 g (80% yield) of product with an e.e. of 99.5%. 

In an alternate process, frozen cells of *S. paucimobilis* SC 16113 were used with XAD-16 hydrophobic resin (50 g/L) adsorbed substrate at 10 g/L concentration. In this process, an average reaction yield of 85% and an e.e. of >99% were obtained for alcohol **68**. At the end of the biotransformation, the reaction mixture was filtered on a 100 mesh (150 μm) stainless steel screen, and the resin retained by the screen was washed with water. The product was then desorbed from the resin with acetonitrile and crystallized in 75% overall yield with a 99.8% e.e.[98].

### 2.18. Penem and Carbapenem: Enzymatic Preparation of (R)-1,3-Butanediol and (R)-4-Chloro-3-Hydroxybutonoate

**Figure 18:**
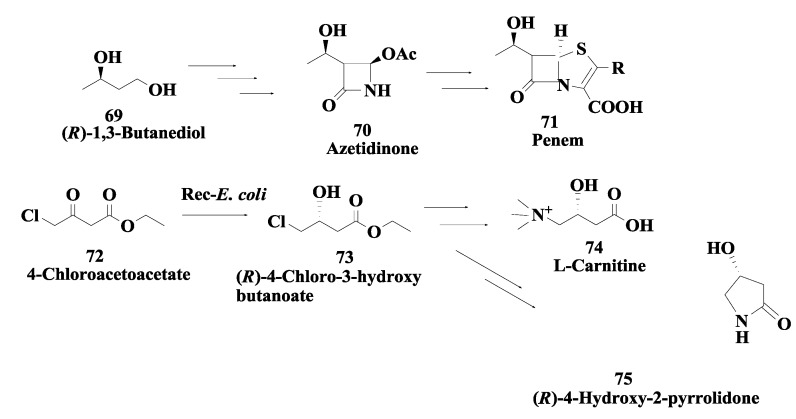
Penem and carbapenem: Enzymatic preparation of (*R*)-1,3-butanediol and (*R*)-4-chloro-3-hydroxybutonoate.

(*R*)-1,3-Butanediol **69** (Figure 18) is a key starting material of azetidinone derivatives **70**, which are key chiral intermediates for the synthesis of penem **71** and carbapenem antibiotics [99]. From a microbial screen*,* the *Candida parapsilosis* strain IFO 1396 was identified which produced (*R*)-1,3-butanediol from the racemate. The (*S*)-1,3-butanediol oxidizing enzyme (CpSADH) which produced (*R*)-1,3-butanediol from the racemate was cloned in *Escherichia coli*. The recombinant culture catalyzed the enantioselective oxidation of secondary alcohols and also catalyzed the asymmetric reduction of aromatic and aliphatic ketones to their corresponding (*S*)-secondary alcohols. Using the recombinant enzyme, (*R*)-1,3-butanediol was produced in 97% yield and 95% e.e. using 150 g/L input of the racemate. Recombinant enzyme (CpSADH) was also used for reduction of ethyl 4-chloroacetoacetate **72** to produce ethyl-(*R*)-4-chloro-3-hydroxybutonoate **73** in 95% yield and 99% e.e. using 36 g/L substrate input. Isopropanol was used to regenerate the NADH required for this reduction. Ethyl-(*R*)-4-chloro-3-hydroxybutonoate is useful for the synthesis of L-carnitine **74** and (*R*)-4-hydroxyl pyrrolidone **75** [100,101]).

### 2.19. Integrin Receptor Agonist: Enzymatic Preparation of (R)-Allylic Alcohol

**Figure 19:**
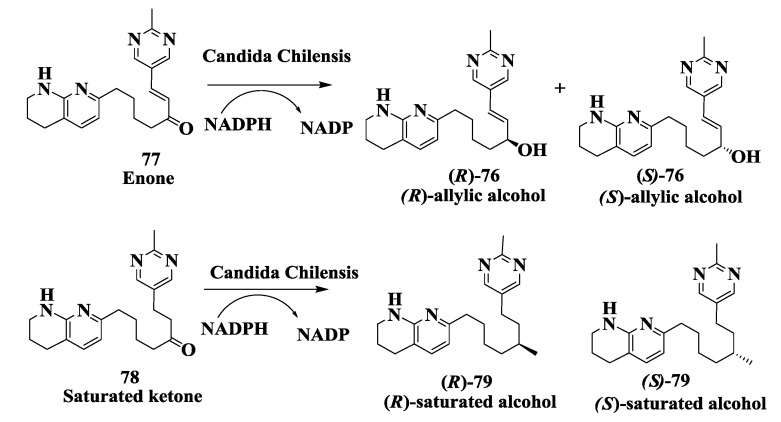
Integrin receptor agonist: Enzymatic preparation of (*R*)-allylic alcohol.

(*R*)-allylic alcohol **76** (Figure 19) was required as an intermediate for the synthesis of a desired monanoic derivate useful as an integrin receptor antagonist for the inhibition of bone desorption and treatment of osteoporosis [102]. A pilot scale whole cell process was developed for the enantioselective 1,2-reduction of prochiral alpha,beta-unsaturated ketone **77** to (*R*) allylic alcohol, (*R*)-**76** using *Candida chilensis* [103]. Initial development showed high enantiomeric excess (>95%) but low product yield (10%). Further process development, using a combination of statistically designed screening and optimization experiments, improved the desired alcohol yield to 90%. The fermentation growth stage, particularly medium composition and growth pH, had a significant impact on the bioconversion while process characterization identified diverse challenges including the presence of multiple enzymes, substrate/product toxicity, and biphasic cellular morphology. Manipulating the fermentation media allowed control of the whole cell morphology to a predominantly unicellular broth, away from the viscous pseudohyphae, which were detrimental to the bioconversion. The activity of a competing enzyme, which produced the undesired saturated ketone **78** and (*R*)-saturated alcohol 79, was minimized to < or =5% by controlling the reaction pH, temperature, substrate concentration, and biomass level. Despite the toxicity effects limiting the volumetric productivity, a reproducible and saleable process was demonstrated at pilot scale with high enantioselectivity (e.e. > 95%) and overall yield greater than 80% [104]. The whole cell approach proved to be a valuable alternative to chemical reduction routes. 

### 2.20. NK1 Receptor Antagonists: Enzymatic Synthesis of (S)-3,5-Bistrifluoromethylphenyl Ethanol

**Figure 20:**
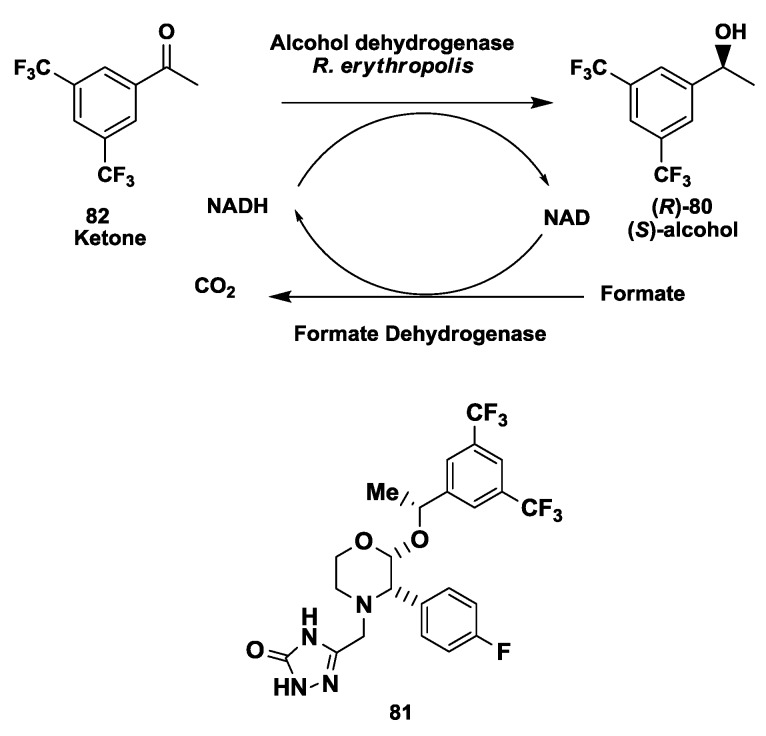
NK1 receptor antagonists: Enzymatic synthesis of (*S*)-3,5-bistrifluoromethylphenyl ethanol.

The synthesis of (*S*)-3,5-bistrifluoromethylphenyl ethanol, (*S*)-**80**, (Figure 20), an intermediate for the synthesis of NK-1 receptor antagonists **81** [104] was demonstrated from a ketone **82** via asymmetric enzymatic reduction process [105]. The isolated enzyme alcohol dehydrogenase from *Rhodococcus erythropolis* reduced the poorly water soluble substrate with an excellent enantiomeric excess (>99.9%) and good conversion (>98%). The optimized process was demonstrated up to pilot scale using concentration (390 mM) using a easy isolation process achieving overall isolation yields (>90%). Process improvements at preparative scale, demonstrated increase in the substrate input to 580 mM achieving a space time yield of 260 g/L/day [105]. 

## 3. Enzymatic Preparation of Chiral Amino Acids

The reductive amination of α-keto acids using amino acid dehydrogenases to be one of the most useful methods because the enzymes have good stability, broad substrate specificity and very high enantioselectivity and can be used at high substrate concentrations as keto acids are soluble in aqueous system. The reductive aminations process coupled to an enzymatic cofactor regeneration system are most prominent method for preparation of chiral amino acids. For most enzymes, the required cofactor is NADH but NADPH is required in some cases. Yeast formate dehydrogenase is commonly used for NADH regeneration and glucose dehydrogenase usually from *Bacillus* species may be used for either NADH or NADPH regeneration. There are excellent reviews on the amino acid dehydrogenases and examples of their synthetic utilities [106,107,108,109].

### 3.1. Tigemonam: Enzymatic Synthesis of (S)-β-Hydroxyvaline

**Figure 21:**
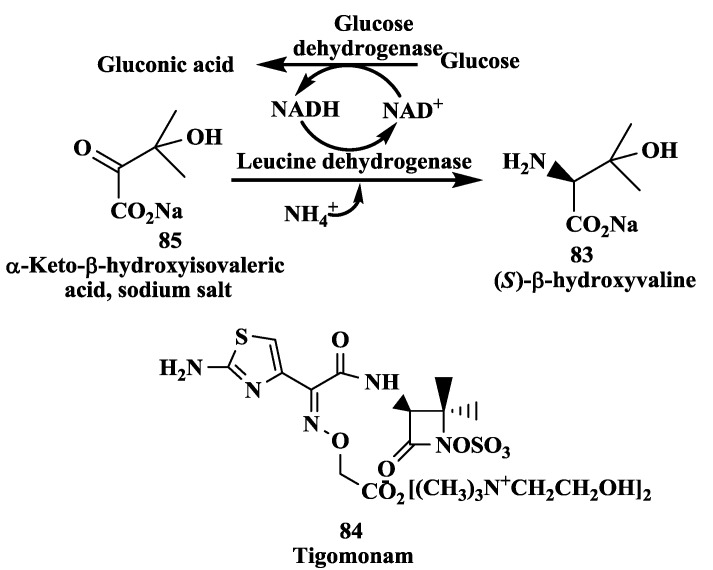
Tigemonam: Enzymatic synthesis of (*S*)-β-hydroxyvaline.

(*S*)-β-hydroxyvaline **83** (Figure 21), is a key chiral intermediate required for the total synthesis of orally active monobactam [110], Tigemonam **84**. Chiral amino acids have been made from corresponding keto acids by reductive amination process [111]. The synthesis of (*S*)-β-hydroxyvaline **83** from α-keto-β-hydroxyisovalerate **85** by reductive amination using leucine dehydrogenase from *Bacillus sphaericus* ATCC 4525 has been demonstrated [112]. The NADH required for this reaction was regenerated by either formate dehydrogenase from *Candida boidinii* or glucose dehydrogenase from *Bacillus megaterium*. The required substrate **85** was generated either from α-keto-β-bromoisovalerate or its ethyl esters by hydrolysis with sodium hydroxide *in situ.* In this process, an overall reaction yield of 98% and an enantiomeric excess of 99.8% were obtained for the L-β-hydroxyvaline **83**.

### 3.2. Atazanavir: Enzymatic Synthesis of (S)-Tertiary-Leucine

**Figure 22:**
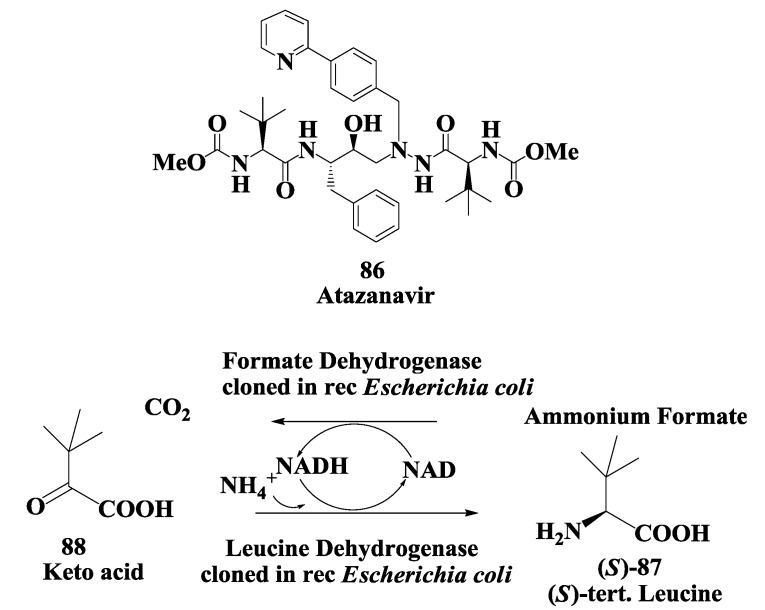
Atazanavir (anti-viral agent): Enzymatic synthesis of (*S*)-tertiary-leucine.

Atazanavir **86** is an acyclic aza-peptidomimetic, a potent HIV protease inhibitor [53,54]. Synthesis of atazanavir required (*S*)-tertiary leucine **87** (Figure 22). An enzymatic reductive amination of ketoacid **88** to amino acid **87** by recombinant *Escherichia coli* expressing leucine dehydrogenase from *Thermoactinimyces intermedius* has been demonstrated. The reaction required ammonia and NADH as a cofactor. NAD produced during the reaction was converted back to NADH using recombinant *Escherichia coli* expressing formate dehydrogenase from *Pichia pastoris*. A reaction yield of >95% with an e.e. of >99.5% was obtained for **87** at 100 g/L substrate [113]. Leucine dehydrogenase from *Bacillus* strain has also been cloned and expressed and used in reductive amination process [114,115].

### 3.3. Vanlev: Enzymatic Synthesis of (S)-6-Hydroxynorleucine

**Figure 23:**
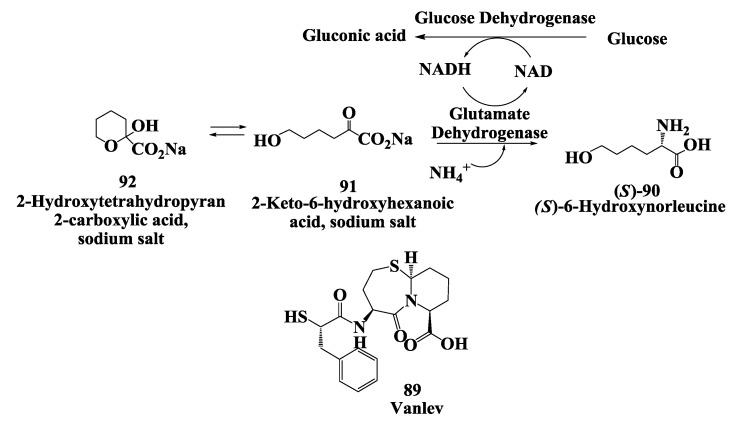
Vanlev: Enzymatic synthesis of (*S*)-6-hydroxynorleucine by reductive amination.

**Figure 24:**
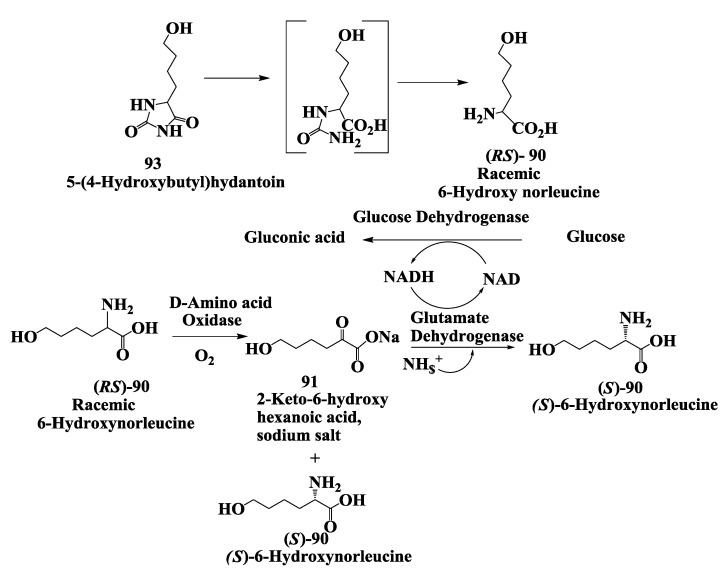
Vanlev: Enzymatic conversion of racemic 6-hydroxy norleucine to (*S*)-6-hydroxymorleucine.

Vanlev **89** (Figure 23) is an antihypertensive drug which acts by inhibiting angiotensin-converting enzyme (ACE) and neutral endopeptidase (NEP) [116]. (*S*)-6-Hydroxynorleucine **90** is a key intermediate in the synthesis of Vanlev. The synthesis and complete conversion of 2-keto-6-hydroxyhexanoic acid **91** to (*S*)-6-hydroxynorleucine **90** was demonstrated by reductive amination using beef liver glutamate dehydrogenase [117]. As depicted, compound **91**, in equilibrium with 2-hydroxytetrahydropyran-2-carboxylic acid sodium salt **92**, was converted to **90**. The reaction requires ammonia and NADH. NAD produced during the reaction was recycled to NADH by the oxidation of glucose to gluconic acid using glucose dehydrogenase from *Bacillus megaterium*. The reaction was complete in about 3 h at 100 g/L substrate input with a reaction yields of 92% and e.e. of 99.8% for (*S*)-6-hydroxynorleucine. The synthesis and isolation of keto acid **91** required several steps. In a second, more convenient process the ketoacid was prepared by treatment of racemic 6-hydroxy norleucine **90** [produced by hydrolysis of 5-(4-hydroxybutyl) hydantoin **93**] with (*R*)-amino acid oxidase (Figure 24) After the e.e. of the unreacted (*S*)-6-hydroxynorleucine had risen to 99.8%, the reductive amination procedure was used to convert the mixture containing the 2-keto-6-hydroxyhexanoic acid entirely to (*S*)-6-hydroxynorleucine in 97% yield with 99.8% e.e. from racemic 6-hydroxynorleucine at 100 g/L substrate input [117]. The (*S*)-6-hydroxynorleucine prepared by the enzymatic process was converted chemically to Valev **89** [118].

### 3.4. Vanlev: Enzymatic Synthesis of Allysine Ethylene Acetal

**Figure 25:**
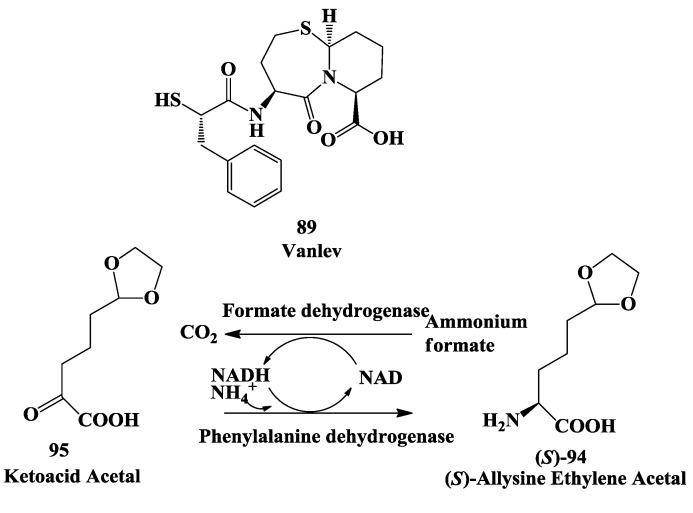
Vanlev: Enzymatic synthesis of allysine ethylene acetal.

(*S*)-2-Amino-5-(1,3-dioxolan-2-yl)-pentanoic acid [(*S*)-allysine ethylene acetal] **94** (Figure 25) is one of three building blocks used in an alternative synthesis of Vanlev **89**. Synthesis of **94** was demonstrated by reductive amination of ketoacid acetal **95** using phenylalanine dehydrogenase [PDH] from *Thermoactinomyces intermedius* [119]. The reaction required ammonia and NADH; NAD produced during the reaction was recyled to NADH by the oxidation of formate to CO2 using formate dehydrogenase [FDH]. *T. intermedius* PDH was cloned and expressed in *Escherichia coli* and recombinant culture was used as a source of PDH. Expression of *T. intermedius* PDH in *P. pastoris*, inducible by methanol, allowed generation of both enzymes in a single fermentation as methanol grown cells of *P. pastoris* also contained formate dehydrogease. A total of 197 kg of **94** was produced in three 1,600-L batches using a 5% concentration of substrate **95** with an average yield of 91 M % and e.e. >98% [119]. (*S*)-allysine ethylene acetal was converted to Vanlev **89** [118].

### 3.5. Saxagliptin: Enzymatic Reductive Amination of 2-(3-Hydroxy-1-Adamantyl)-2-Oxoethanoic Acid

Dipeptidyl peptidase 4 (DPP-4) is a ubiquitous proline-specific serine protease responsible for the rapid inactivation of incretins, including glucagon-like peptide 1 (GLP-1) and glucose-dependent insulinotropic peptide. To alleviate the inactivation of GLP-1, inhibitors of DPP-IV are being evaluated for their ability to provide improved control of blood glucose for diabetics [120,121,122,123]. Januvia developed by Merck is a marketed DPP4 Inhibitor [122].

**Figure 26:**
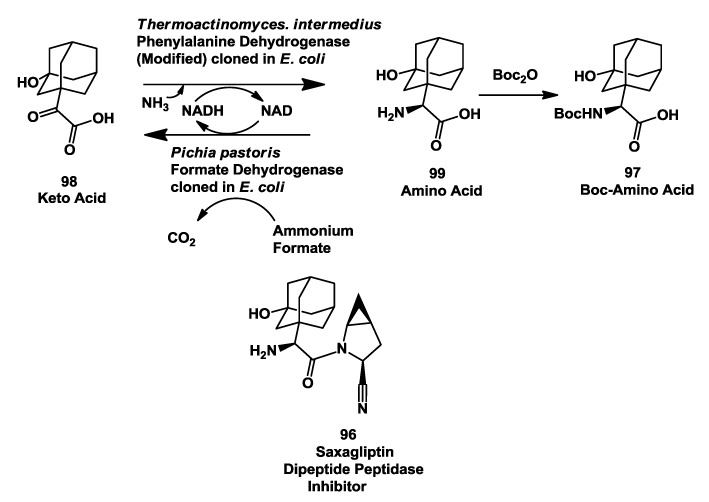
Saxagliptin: Enzymatic reductive amination of 2-(3-hydroxy-1-adamantyl)-2-oxoethanoic acid.

Saxagliptin **96** [121,122] (Figure 26), a DPP-IV inhibitor developed by Bristol-Myers Squibb and now approved for type 2 diabetic treatment by Food and Drug administration, requires (*S*)-*N*-boc-3-hydroxyadamantylglycine **97** as an intermediate. A process for conversion of the keto acids **98** to the corresponding amino acid **99** using (*S*)-amino acid dehydrogenases was developed. A modified form of a recombinant phenylalanine dehydrogenase cloned from *Thermoactinomyces intermedius* and expressed in *Pichia pastoris* or *Escherichia coli* was used for this process. NAD produced during the reaction was recycled to NADH using formate dehydrogenase. The modified phenylalanine dehydrogenase contains two amino acid changes at the C-terminus and a 12 amino acid extension of the C-terminus [124].

Production of multi-kg batches was originally carried out with extracts of *Pichia pastoris* expressing the modified phenylalanine dehydrogenase from *Thermoactinomyces intermedius* and endogenous formate dehydrogenase. The reductive amination process was further scaled up using a preparation of the two enzymes expressed in single recombinant *E. coli*. The amino acid **99** was directly protected as its boc derivative without isolation to afford intermediate. Yields before isolation were close to 98% with 100% e.e. [124]. 

Reductive amination was also conducted using cell extracts from *E.coli* strain SC16496 expressing PDHmod and cloned FDH from *Pichia pastoris*. Cell extracts after polyethyleneamine treatment, clarification and concentration were used to complete the reaction in 30 h with >96% yield and >99.9% e.e. of product **99**. This process has now been used to prepare several hundred kg of boc-protected amino acid **97** to support the development of Saxagliptin [124]. 

### 3.6. Enzymatic Synthesis of (S)-Neopentylglycine

**Figure 27:**
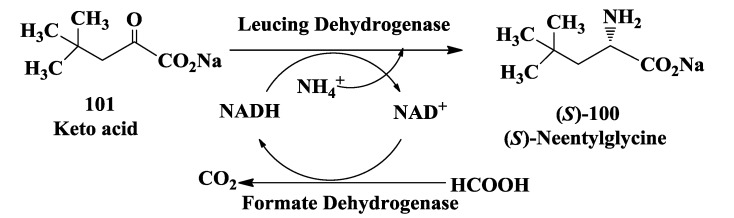
Enzymatic synthesis of (*S*)-neopentylglycine.

The enantioselective synthesis of (*S*)-neopentylglycine **100** (Figure 27) has been developed by Groeger *et al.* [125]. Recombinant whole cell containing leucine dehydrogenase and formate dehydrogenase was used in the reductive amination of the corresponding α-keto acid **101**. The desired (*S*)-neopentylglycine was obtained with >95% conversion and a high enantioselectivity of >99% e.e. at substrate concentrations of up to 88 g/L. Spiroheterocyclic compounds [morpholine-4-carboxylic acid amides of heterocyclic cyclohexylalanine and neopentylglycine derivatives and their analogs] are useful as reversible inhibitors of cysteine proteases such as cathepsin S useful in the treatment of variety of autoimmune diseases [126].

### 3.7. Glucogen like Peptide: Enzymatic Deracemization Racemic Amino Acid to (S)-Amino Acid

**Figure 28:**
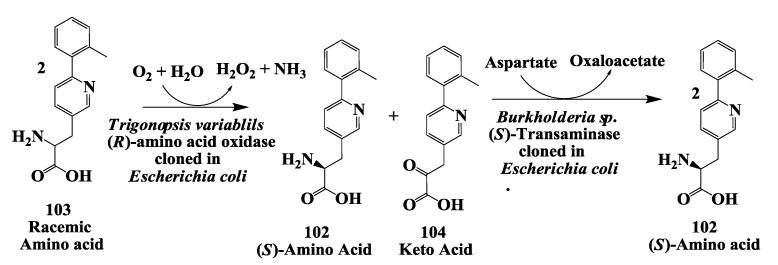
Glucogen like peptide: The (*S*)-amino-3-[3-{6-(2-methylphenyl)}pyridyl]-propionic acid.

The (*S*)-amino-3-[3-{6-(2-methylphenyl)}pyridyl]-propionic acid 102 (Figure 28) is a key intermediate required for synthesis of GLP-1 mimics or GLP-1 receptor modulators. Such receptor modulators are potentially useful for the treatment of type II diabetes treatment [127,128]. 

(*S*)-Amino-3-[3-{6-(2-methylphenyl)}pyridyl]-propionic acid was prepared by enzymatic deracemization process [129] in 72% isolated yield with >99.4% e.e. from racemic amino acid **103** using combination of two enzymes (*R*)-amino acid oxidase from *Trigonopsis variabilis* expressed in *Escherichia coli* and (*S*)-aminotransferase from *Sporosarcina ureae* cloned and expressed in *Escherichia coli*. (*S*)-aspartate was used as amino donor. A (*S*)-aminotransferase was also purified from a soil organism identified as *Burkholderia sp.* and cloned and expressed in *Escherichia coli* and used in this process [131]. In enzymatic process racemic amino acid was first treated with (*R*)-amino acid oxidase for 4 h to convert racemic amino acid to mixture of (*S*)-amino acid and keto acid **104**. Subsequently in the same reaction mixture (*S*)-aminotransferase was charged to convert keto acid **104** to (*S*)-amino acid **102** to get 85% yield at the end of the biotransformation process. This process was scaled up to 100 L scale at a substrate input of 1.5 kg.

In an alternate process, the enzymatic dynamic resolution of racemic amino acid **103** was also demonstrated. (*R*)-selective oxidation with celite-immobilized (*R*)-amino acid oxidase from *Trigonopsis variabilis* expressed in *Escherichia coli* in combination with chemical imine reduction with borane-ammonia gave a 75% in process yield and 100 e.e. of (*S*)-amino acid **102** [129]. 

### 3.8. Preparation of (R)-Amino Acid

**Figure 29:**
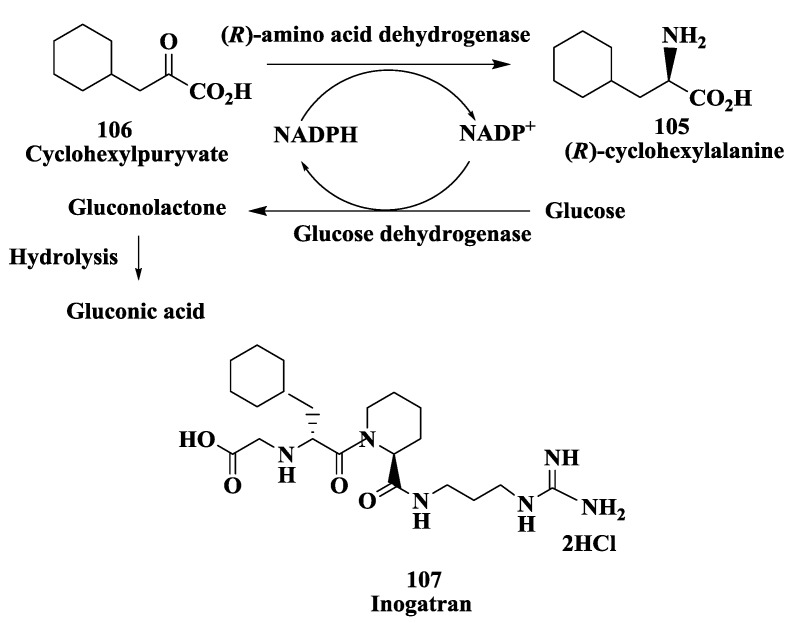
Thrombin inhibitor inogatran: Enzymatic synthesis of (*R*)-cyclohexylalanine.

(*R*)-Amino acids are increasingly becoming important building blocks in the production of pharmaceuticals and fine chemicals, and as chiral directing auxiliaries and chiral synthons in organic synthesis [130,131]. Using both rational and random mutagenesis, Rozzell and Novick [132] have created the broad substrate range, nicotinamide cofactor dependent, and highly stereoselective (*R*)-amino acid dehydrogenase. This new enzyme is capable of producing (*R*)-amino acids via the reductive amination of the corresponding 2-keto acid with ammonia. This biocatalyst was the result of three rounds of mutagenesis and screening performed on the enzyme *meso*-diaminopimelate (*R*)-dehydrogenase from *Corynebacterium glutamicum*. The first round targeted the active site of the wild-type enzyme and produced mutants that were no longer strictly dependent on the native substrate. The second and third rounds produced mutants that had an increased substrate range including straight- and branched-aliphatic amino acids and aromatic amino acids. The very high selectivity toward the (*R*)-enantiomer (95% to >99% e.e.) was shown to be preserved three rounds of mutagenesis and screening [132]. This new enzyme was active against variety of amino acids could complement and improve upon current methods for (*R*)-amino acid synthesis. The synthesis of (*R*)-cyclohexylalanine 105 (Figure 29) was developed by reductive amination of cyclohexylpyruvate **106** to yield (*R*)-**105** in 98% yield and >99% e.e. (*R*)-105 is a potential chiral intermediate for the synthesis of thrombin inhibitor Inogatran **107** [133].

The deracemisation of DL-amino acids using L-amino acid oxidase from *Proteus myxofaciens* and amine-boranes as chemical reducing agents has been investigated. Amine-boranes were found to be of particular interest in terms of reactivity and chemoselectivity compared to sodium borohydride and cyanoborohydride. Starting from the racemic amino acids, a range of D-amino acids were prepared in yields of up to 90% and e.e. >99% [134].

### 3.9. Calcitonin Gene-Related Peptide Receptors (Antimigraine Drugs): Enzymatic Deracemization Process

**Figure 30:**
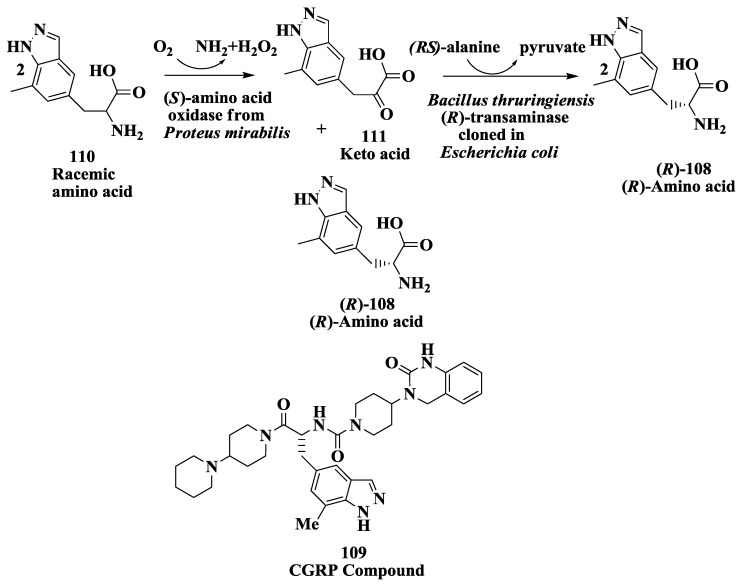
Calcitonin gene-related peptide receptors (antimigraine drugs): Enzymatic preparation of (*R*)-2-amino-3-(7-methyl-1*H*-indazol-5-yl)propanoic acid.

The (*R*)-amino acid (*R*)-2-amino-3-(7-methyl-1 H-indazol-5-yl)propanoic acid (*R*)-**108**, (Figure 30) is a key intermediate needed for synthesis of antagonists of calcitonin gene-related peptide receptors **109** [135] Such antagonists are potentially useful for the treatment of migraine and other maladies [135,136]. 

(*R*)-Amino acid 108 was prepared in 68% isolated yield with >99% e.e. from racemic amino acid **110** using (*S*)-amino acid oxidase from *Proteus mirabilis* expressed in *Escherichia coli* in combination with a commercially available (*R*)-transaminase using (*R*)-alanine as amino donor [137]. The (*R*)-enantiomer was also prepared in 79% isolated yield with >99% e.e. from the corresponding keto acid **111** using the (*R*)-transaminase with racemic alanine as the amino donor. The rate and yield of this reaction could be accelerated by addition of lactate dehydrogenase (with NAD^+^, formate and formate dehydrogenase to regenerate NADH) to remove the inhibitory pyruvate produced during the reaction. A (*R*)-transaminase was identified and purified from a soil organism identified as *Bacillus thuringiensis* and cloned and expressed in *Escherichia coli*. The recombinant (*R*)-transaminase was very effective for the preparation of (*R*)-**108** and gave a nearly complete conversion of **111** to (*R*)-**108** without the need for additional enzymes for pyruvate removal [137].

### 3.10. Corticotropin Releasing Factor (CRF)-1 Receptor Antagonist: Enzymatic Resolution by Transaminase

**Figure 31:**
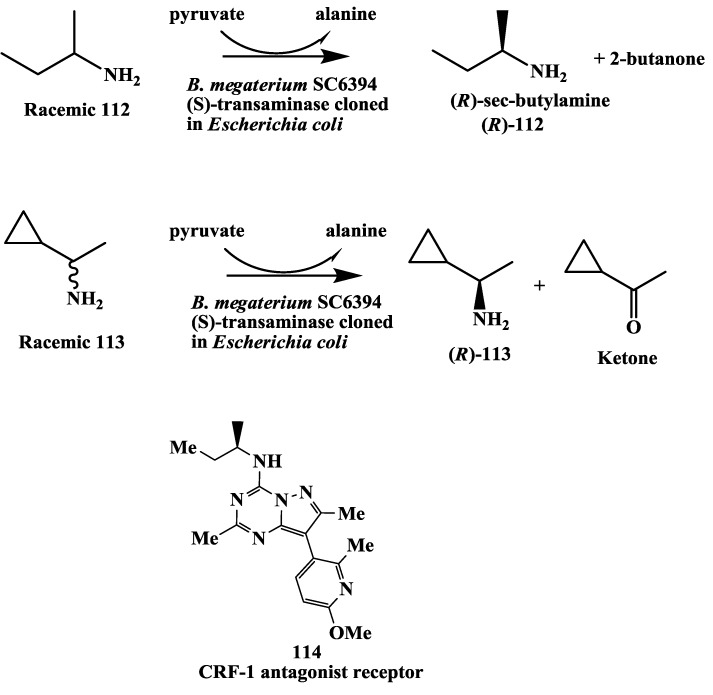
Corticotropin releasing factor (CRF)-1 receptor antagonist: Enzymatic synthesis of (*R*)-1-cyclopropylethylamine and (*R*)-*sec*-butylamine.

(*R*)-amines synthesis for Anxiety and depression are psychiatric disorders that constitute a major health concern worldwide. While numerous marketed treatments exits for both disorders, there continue to be need agents which may have increased efficacy and/or reduced side-effect profiles [138,139,140]. CRF-1 receptor antagonists have been proposed as novel pharmacological treatments for depression, anxiety and stress disorders [138,139,140,141]. (*R*)-sec-butylamine **112** and (*R*)-1-cyclopropylethylamine **113** (Figure 31) are key chiral intermediates for the synthesis of CRF-1 receptor antagonists such as **114** [141,142]. 

We have developed enzymatic resolution process for the preparation of (*R*)-sec-butylamine and (*R*)-1-cyclopropylethylamine [143]. Screening was carried out to identify strains useful for the preparation of (*R*)-1-cyclopropylethylamine and (*R*)-*sec*-butylamine from the racemic amines with an (*S*)-specific transaminase. Several *Bacillus megaterium* strains as well as several soil isolates were found to have the desired activity for the resolution of the racemic amines to give the (*R*)-enantiomers. Using an extract of the best strain, *Bacillus megaterium* SC6394, the reaction was shown to be a transamination requiring pyruvate as amino acceptor and pyridoxal phosphate as a cofactor. Initial batches of both amines were produced using whole cells of *Bacillus megaterium* SC6394. The transaminase was purified to homogeneity to obtain N-terminal as well as internal amino acid sequences. The sequences were used to design polymerase chain reaction (PCR) primers to enable cloning and expression of the transaminase in *Escherichia coli* SC16578. In contrast to using *Bacillus megaterium* process, pH control and aeration were not required for the resolution of *sec*-butylamine and an excess of pyruvate was not consumed by the recombinant cells. The resolution of *sec*-butylamine (0.68 M) using whole cells of *Escherichia coli* SC16578 was scaled up to give (*R*)-*sec*-butylamine 1/2 H_2_SO_4_ in 46.6% isolated yield with 99.2% *e.e*. An alternative isolation procedure was also used to isolate (*R*)-*sec*-butylamine as the free base. Using the same recombinant (*S*)-tansaminase, (*R*)-1-cyclopropylethylamine was obtained in 42% isolated yield (theoretical max. 50%) and 99% e.e. [143].

## 4. Conclusions

The production of single enantiomers of drug intermediates is increasingly important in the pharmaceutical industry. Biocatalysis provides organic chemists an alternate opportunity to prepare pharmaceutically important chiral compounds. The examples presented in this review are only from a few selected articles for synthresis of chiral alcohols and unnatural amino acids. Different types of biocatalytic reactions are capable of generating a wide variety of chiral compounds useful in the development of drugs. The use of hydrolytic enzymes such as lipases, esterases, proteases, dehalogenases, acylases, amidases, nitrilases, epoxide hydrolases, and decarboxylases for the resolution of variety of racemic compounds and in the asymmetric synthesis of enantiomerically enriched chiral compounds. Dehydrogenases and aminotransferases has been successfully used along with cofactors and cofactor regenerating enzymes for the synthesis of chiral alcohols, aminoalcohols, amino acids and amines. Aldolases and decarboxylases have been effectively used in asymmetric synthesis by aldol condensation and acyloin condensation reactions. Monoxygenases have been used in enantioselective and regioselective hydroxylation, epoxidation, sulfoxidation and Baeyer-Villiger reactions. Dioxygenases have been used in the chemo-enzymatic synthesis of chiral diols. Enzymatic deracemization, dynamic resolution and stereoinversion, to achieve >50% yield and high e.e. by combination of chemo- and/or biocatalysts in sequential reactions or by a single biocatalyst. In the course of the last decade, progress in biochemistry, protein chemistry, molecular cloning, random and site-directed mutagenesis, directed evolution of biocatalysts under desired process conditions has opened up unlimited access to a variety of enzymes and microbial cultures as tools in organic synthesis. Future of bicatalysis for synthesis of chiral compounds looks very promising.
